# Lipoprotein Lipase and PPAR Alpha Gene Polymorphisms, Increased Very-Low-Density Lipoprotein Levels, and Decreased High-Density Lipoprotein Levels as Risk Markers for the Development of Visceral Leishmaniasis by *Leishmania infantum*

**DOI:** 10.1155/2014/230129

**Published:** 2014-08-27

**Authors:** Márcia Dias Teixeira Carvalho, Diego Peres Alonso, Célia Maria Vieira Vendrame, Dorcas Lamounier Costa, Carlos Henrique Nery Costa, Guilherme Loureiro Werneck, Paulo Eduardo Martins Ribolla, Hiro Goto

**Affiliations:** ^1^Laboratório de Soroepidemiologia e Imunobiologia, Instituto de Medicina Tropical de São Paulo, Universidade de São Paulo, Avenida Dr. Enéas de Carvalho Aguiar 470, Prédio II, 4° Andar, 05403-000 São Paulo, SP, Brazil; ^2^Departamento de Parasitologia, Instituto de Biociências, Universidade Estadual Paulista, 18618-970 Botucatu, SP, Brazil; ^3^Instituto de Doenças Tropicais Natan Portella, Universidade Federal do Piauí, 64001-450 Teresina, PI, Brazil; ^4^Departamento de Epidemiologia, Instituto de Medicina Social, Universidade do Estado do Rio de Janeiro, 20550-900 Rio de Janeiro, RJ, Brazil; ^5^Instituto de Estudos em Saúde Coletiva, Universidade Federal do Rio de Janeiro, 21941-598 Rio de Janeiro, RJ, Brazil; ^6^Departamento de Medicina Preventiva, Faculdade de Medicina, Universidade de São Paulo, 01246-903 São Paulo, SP, Brazil

## Abstract

In visceral leishmaniasis (VL) endemic areas, a minority of infected individuals progress to disease since most of them develop protective immunity. Therefore, we investigated the risk markers of VL within nonimmune sector. Analyzing infected symptomatic and, asymptomatic, and noninfected individuals, VL patients presented with reduced high-density lipoprotein cholesterol (HDL-C), elevated triacylglycerol (TAG), and elevated very-low-density lipoprotein cholesterol (VLDL-C) levels. A polymorphism analysis of the lipoprotein lipase (LPL) gene using HindIII restriction digestion (*N* = 156 samples) (H+ = the presence and H− = the absence of mutation) revealed an increased adjusted odds ratio (OR) of VL versus noninfected individuals when the H+/H+ was compared with the H−/H− genotype (OR = 21.3; 95% CI = 2.32–3335.3; *P* = 0.003). The H+/H+ genotype and the H+ allele were associated with elevated VLDL-C and TAG levels (*P* < 0.05) and reduced HDL-C levels (*P* < 0.05). An analysis of the L162V polymorphism in the peroxisome proliferator-activated receptor alpha (PPARα) gene (*n* = 248) revealed an increased adjusted OR when the Leu/Val was compared with the Leu/Leu genotype (OR = 8.77; 95% CI = 1.41–78.70; *P* = 0.014). High TAG (*P* = 0.021) and VLDL-C (*P* = 0.023) levels were associated with susceptibility to VL, whereas low HDL (*P* = 0.006) levels with resistance to infection. The mutated LPL and the PPARα Leu/Val genotypes may be considered risk markers for the development of VL.

## 1. Introduction

Visceral leishmaniasis (VL) is a systemic disease that is prevalent in tropical and subtropical regions. VL is caused by various species of protozoa within the genus* Leishmania* and of the* Leishmania donovani* complex, including* Leishmania infantum *(previously referred to as* L. chagasi*), in the new world [1]. In a VL-endemic area, the most infected individuals remain asymptomatic. Individuals with infection may present with mild symptoms and a minority of infected individuals, approximately 10–20%, progress to active severe disease that is characterized by fever, hepatosplenomegaly, pancytopenia, hypergammaglobulinemia, and severe weight loss [2, 3]. However, the factors that are associated with susceptibility to infection progression are not well known.

The immune system has been the natural focus of previous studies that assessed the risk markers for VL development. Most studies have focused on genes that are related to the immune system. In studies with approaches that were based mainly on family groups with VL infection, the SLC11A1 (formerly called as the natural resistance-associated macrophage protein-1, NRamp-1) gene on chromosome 2q35 [4, 5], interleukin-4/interleukin-9 [6], the TNF locus [7], a locus on chromosome 22q12 [8, 9], IL-18 [10], and IL-1beta [11] polymorphisms have been associated with VL infection. However, in their 2009 review [12], Blackwell et al. suggested that most of these polymorphisms had marginal effects on infection progression. Recently, the IL-10 819 C/T genotype was associated with susceptibility to VL development in Iran [13]. In well-powered population-based studies, the CXCR2 gene [14] and DLL1, which encode delta-like 1, the ligand for Notch 3 [15], were associated with susceptibility to VL development but not SLC11A1 in India [16]. More recently, another well-powered population-based study revealed a consistent contribution of the HLA-DRB1-HLA-DQA1 HLA class II region to VL susceptibility in the Indian subcontinent and Brazil [17]. However, the role of these genetic determinants in the pathogenesis of VL was not addressed in these studies.

In* L. infantum* infection, protective immunity develops in the majority of individuals who reside in VL-endemic areas [2]. During active disease, immune derangement occurs; however, the immune system remains highly activated [3]. Therefore, nonspecific factors may play important roles in infections by parasites that can efficiently evade effective immune responses. Furthermore, a study has indicated that factors not related to acquired cell-mediated immunity may explain the progression of VL [18]. Therefore, in this study, we focused on nonimmune sector, that is, lipid metabolism.

Several studies have reported on lipid alterations in human VL cases [19–22]. In this study, we initially analyzed serum-lipid profiles in a large number of patients with clinical manifestations of VL. We found high triacylglycerol (TAG) levels, high very-low-density lipoprotein cholesterol (VLDL-C) levels, and low high-density lipoprotein cholesterol (HDL-C) levels in infected symptomatic individuals. These alterations may have resulted from the inflammatory infectious process; however, we investigated the possible role of these lipoprotein fractions in susceptibility to* L. infantum* infection because previous results have suggested that amastigotes are largely dependent on protein and lipids as an energy source within host cells [23]. In addition to the analysis of lipoprotein fraction alterations, we extended the study to analyze the factors that modulate TAG and HDL levels. These additional factors included lipoprotein lipase (LPL), which hydrolyzes TAG from TAG-rich lipoprotein particles in plasma [24] and reduces the uptake of a TAG-rich artificial emulsion [25], apolipoprotein E (apoE), which mediates the binding of lipoprotein particles to receptors in the liver that mediate their clearance [26], and peroxisome proliferator-activated receptor alpha (PPARα), which is involved in lipid metabolism, including roles in oxidation pathways, the uptake and transport of fatty acids, and lipoprotein synthesis routes [27]. Therefore, we focused on LPL, apoE, and PPARα gene polymorphisms as candidates for genetic risk markers for disease development in* L. infantum* infection. LPL gene polymorphisms were searched using HindIII and PVuII restriction enzymes known to reveal mutated genes that correlate with TAG level alteration and the former also with HDL level alteration [28, 29]. We found that the H+/H+ genotype and the H+ allele, which were identified using HindIII restriction in the LPL gene, were more frequent in infected symptomatic individuals and were notably associated with high levels of VLDL-C, TAG, and HDL-C. The apoE gene is presented in three main isoforms (apoE2, apoE3, and apoE4) that are coded by three common alleles (E2, E3, and E4) [26]. We found that the E3/E3 genotype and the E3 allele were more frequent in infected symptomatic individuals than in infected asymptomatic or noninfected individuals, and these factors were associated with high levels of VLDL cholesterol and TAG. We found that the Leu/Val genotype and the Val allele of the L162V polymorphism in the PPARα gene were more frequent in infected symptomatic individuals than in infected asymptomatic or noninfected individuals but with no apparent association with alterations in lipoprotein levels.

We hypothesized that certain lipid fractions may play a role in the development of* L. infantum* infection. In this study, we found that high levels of triglycerides and VLDL and low levels of HDL favor the development of active disease. Therefore, we suggest that related lipoprotein lipase and apolipoprotein E genotypes that lead to alterations in these lipoprotein levels as well as the presence of the PPARα L162V allele may be susceptibility factors and markers of VL development.

## 2. Materials and Methods

### 2.1. Study Design and Subjects

The individuals who were included in this study were from Teresina, Piaui State, Northeastern Brazil. The inclusion criteria were as follows: unrelated individuals who presented with active VL (referred to as infected symptomatic), healthy individuals who had been infected with* L. infantum *as determined by a positive anti-*Leishmania* (Montenegro) delayed-type hypersensitivity skin test and/or the presence of anti-*Leishmania* antibodies (referred to as infected asymptomatic), and healthy individuals who had no evidence of present or past infection with* L. infantum *as determined by both a negative Montenegro skin test and the absence of specific antibodies. The subjects and materials in this study were similar to those in the previous study by Alonso et al. [30] in which possible ethnic and environmental biases were analyzed and deemed not to be confounding factors.

### 2.2. Ethics Statement

This study was approved by the ethics committees of the participating institutions (Comissão de Ética em Pesquisa da Universidade Federal do Piauí and Comissão de Ética para Análise de Projetos de Pesquisa da Diretoria Clínica do Hospital das Clínicas e da Faculdade de Medicina da Universidade de São Paulo). Written informed consent was obtained from all of the participants or their parents or guardians. Additionally, the samples and data were treated anonymously.

### 2.3. Analysis of Biochemical Parameters

After 12 hours of fasting, venous blood samples were drawn from 81 individuals to measure lipid parameters. The total cholesterol (TC), low-density lipoprotein cholesterol (LDL-C), HDL-C, and TAG concentrations were measured using automated methods (Roche Diagnostics Co., Indianapolis, IN, USA), and the VLDL-C concentrations were calculated (TAG concentration/5) using the Friedewald equation.

### 2.4. Genetic Analysis of the LPL Polymorphism

DNA was extracted from peripheral blood white cells using the GFX Genomic Blood DNA Purification Kit (GE Healthcare, Piscataway, NJ, USA) and subjected to amplification by polymerase chain reaction (PCR) using a Bio-Rad Laboratories DNA Thermal Cycler (Hercules, CA, USA).

The variations were confirmed using a restriction fragment length polymorphism (RFLP) assay in which the PCR-amplified products were digested by HindIII for 156 individuals or by PvuII for 130 individuals. Due to the availability, not all samples were analyzed using both enzymes. One primer set was used to amplify the sequence around a HindIII restriction site in intron 8 (the forward primer was 5′-TTTAGGCCTGAAGTTTCCAC-3′, and the reverse primer was 5′-CTCCCTAGAACAGAAGATC-3′) [31]. The amplified fragment was 1.3 kb in size. The other primer set was obtained from the DNA sequences that flanked a PvuII restriction site in intron 6 (the forward primer was 5′-TAGAGGTTGAGGCACCTGTGC-3′, and the reverse primer was 5′-GTGGGTGAATCACCTGAGGTC-3′) [32]. The amplified fragment was 858 bp long. The amplification of the region that flanked the HindIII site was performed over 33 cycles at 95°C for 1 min, 60°C for 2 min, and 72°C for 2 min. The amplification of the sequence that surrounded the PvuII site was performed over 32 cycles at 95°C for 1 min, 68°C for 2 min, and 72°C for 2 min. The PCR-amplified products were digested with HindIII or PvuII. HindIII yielded fragments that were 600 bp and 700 bp long, and PvuII yielded fragments that were 266 bp and 592 bp long [28].

### 2.5. Genetic Analysis of the apoE Polymorphism

Using extracted DNA, the variations were confirmed by a single-nucleotide polymorphism (SNP) assay in which the PCR-amplified products were analyzed using the SNaPshot Multiplex Kit (Applied Biosystems, Foster City, CA, USA) for 115 individuals. We used one set of primers that were derived from exon 4 in the apoE gene, and PCR was used to amplify a 244 bp region that spanned the E2, E3, and E4 allelic sites (the forward primer was 5′-TAAGCTTGGCACGGCTGTCCAAGGA-3′, and the reverse primer was 5′-ACAGAATTCGCCCCGGCCTGGTACAC-3′) [26]. The amplification of the apoE site was performed with an initial denaturation at 95°C for 2 min, which was followed by 35 cycles at 95°C for 1 min, 60°C for 1 min, and 72°C for 30 s. The SNaPshot Multiplex Kit was used to investigate the presence of the E2, E3, and E4 alleles in the amplified products. Two specific primers for apoE SNPs were utilized (the forward primer was 5′-GCGCGGACATGGAGGACGTG-3′, and the reverse primer was 5′-CCCCCGGCCTGGTACACTGCCAGGC-3′). The SNaPshot Multiplex Kit reactions were performed in the ABI 377 Automatic Sequencer (Applied Biosystems) following the manufacturer's recommendations.

### 2.6. Genotyping of the PPARα L162V Polymorphism

Using extracted DNA, the PPARα gene L162V polymorphism was genotyped by allelic discrimination using TaqMan probes on a custom assay-on-demand basis by Applied Biosystems. A pair of oligos was custom-designed to amplify a fragment that contained the L162V SNP together with different allele-specific fluorescent probes using PCR. Genotyping was performed using a real-time PCR reaction (StepOnePlus Applied Biosystems) according to the following protocol: in a 10 *μ*L reaction, 5 *μ*L of Maxima Probe/ROX qPCR Master Mix (2X) (Fermentas) was mixed with 0.5 *μ*L of TaqMan reagents (a 20X master mix that contained the probes and the pair of oligos) and 4.5 *μ*L of autoclaved Milli-Q water. The reaction was performed following the cycling protocol, initial denaturation for 10 min at 95°C, followed by 40 cycles of denaturation at 95°C for 15 s, and annealing and extension for 60 s at 60°C. After cycling, a graph of allelic discrimination was generated.

### 2.7. Statistical Analysis

The data were expressed as means and standard deviations and compared using ANOVA tests. The Student Newman-Keuls posttest was used for post hoc statistical comparisons among the groups, and the *t*-test was used for statistical comparisons between the groups. The frequencies and comparison distributions of the genotypes and alleles for each polymorphic site were estimated by gene counting. Consistency with the Hardy-Weinberg proportions was tested by the log-likelihood ratio. We used the Pearson *χ* ^2^ test for trends to analyze differences in the frequency of the LPL genotypes and alleles between the outcome groups (infected symptomatic, infected asymptomatic, and noninfected). Associations between levels of lipids and different genotypes and the patient outcomes were expressed as odds ratios (OR) and their respective 95% confidence intervals (95% CI), adjusted for age and gender, estimated by means of exact logistic regression models. Models compared the infected (both symptomatic and asymptomatic) individuals to the noninfected individuals. The levels of lipids were analyzed both as continuous and dichotomous variables. For the dichotomous analyses, the cut-off points used for TC, LDL-C, HDL-C, TAG, and VLDL-C were established by selecting the point of the ROC curve that maximized the sensitivity and specificity for separating noninfected and infected symptomatic individuals. Data were analyzed using Stata statistical software (Stata for Windows 13.0, StataCorp, College Station, TX), Systat version 10 (Point Richmond, Richmond, CA), and GraphPad Prism 3.0 (GraphPad Software Inc., San Diego, CA, USA). A *P* value <0.05 was considered to be significant.

## 3. Results

In this study, using a candidate gene approach to reveal the genetic traits and the resulting phenotypic expressions, that is, lipoprotein levels, we aimed to identify the risk markers for the development of* L. infantum* infection.

The assessment of the lipid fraction levels revealed elevated triacylglycerol (TAG) levels and elevated very-low-density lipoprotein cholesterol (VLDL-C) levels in all of the infected symptomatic individuals compared with the infected asymptomatic and noninfected individuals (*P* < 0.05) (Table 1) (according to the III Brazilian Guidelines on Dyslipidemia [33], the optimal TAG level is <130 mg/dL for individuals up to 20 years of age and <150 mg/dL in individuals who are older than 20 years of age). Because the ideal age-matched endemic controls for this approach could not be obtained due to technical and ethical reasons, the average age (mean ± standard deviation) of the infected symptomatic individuals (*n* = 17; 6.2 ± 7.6 years of age) was significantly lower than the average ages of the infected asymptomatic (*n* = 28; 30.2 ± 12.4 years of age) and noninfected individuals (*n* = 36; 27.9 ± 19.2 years of age). To circumvent bias, we separately examined the individuals who were younger than 20 years of age when the differences between the groups were still maintained (data not shown). Additionally, we observed reduced high-density lipoprotein cholesterol (HDL-C) levels in both the infected symptomatic and asymptomatic individuals compared with the noninfected individuals (*P* < 0.05) (Table 1).

We analyzed the polymorphisms of the LPL, apoE, and PPARα genes. The genotype distributions for all of the polymorphisms were in Hardy-Weinberg equilibrium for all of the groups (Table 2). Significant differences were observed in the frequencies of the polymorphic HindIII-restricted genotypes (H+/H+ versus H+/H−, *P* < 0.05; H+/H+ versus H−/H−, *P* < 0.01) and the HindIII-restricted alleles (H+ versus H−, *P* < 0.01) (Table 1). However, no differences were detected between the groups according to the frequencies of either the genes or the alleles of the PvuII restriction products (Table 2). In the apoE gene, the analysis of the three common apoE alleles (E2, E3, and E4) that code the three main apoE isoforms (apoE2, apoE3, and apoE4) indicated that the E3/E3 genotype was the most frequent, followed by the E3/E4, E2/E3, E2/E4, and E4/E4 genotypes. The E2/E2 genotype was not detected in any of the individuals. In the analysis of the apoE alleles, the E3 allele was the most frequent, followed by the E4 and E2 alleles. Significant differences were observed between the frequency of the E4 allele and that of the E3 allele (*P* < 0.05) (Table 2). We found that the E3/E3 genotype and the E3 allele were more frequent in infected symptomatic individuals than in infected asymptomatic or noninfected individuals (Table 2). In the PPARα gene L162V polymorphism, we found significant differences in the frequencies of the Leu/Leu and Leu/Val (*P* < 0.05) genotypes and the Leu and Val alleles (*P* < 0.01) between the groups (Table 2).

In the analysis of the association between the genotypes and the lipoprotein levels in all of the individuals regardless of the outcome, the TAG and VLDL-C levels were significantly higher in the H+/H+ individuals (*P* < 0.01) (Table 3). The individuals with the H+/H+ genotype had significantly lower HDL-C levels than the individuals with the H+/H− genotype (*P* < 0.05). The serum-lipid levels were measured in only two H−/H− individuals, which was insufficient for the statistical analysis (the means are presented in the legend of Table 3). In the individuals with the P1/P1 genotype, no significant differences in the lipid profiles were observed between the P1/P2 and P2/P2 individuals (Table 3). In the analysis of the apoE genotypes and alleles, we found that the TAG and VLDL-C levels in the individuals with the E2/E3 genotype were significantly higher than those in the E3/E3 and E3/E4 (*P* < 0.05) individuals (Table 3). The serum-lipid data were available in only one E4/E4 individual (all of the variables are shown in the legend of Table 3). The serum-lipid levels were not evaluated in the E2/E4 and E2/E2 individuals. Furthermore, the TAG and VLDL-C levels were significantly higher in the E2 individuals than in the E3 and E4 individuals (*P* < 0.05), but the TC levels were higher in the E3 individuals than in the E2 and E4 individuals (*P* < 0.05) (Table 3). The E3 allele was associated with higher TC levels than with the E2 and E4 alleles; however, the levels were still optimal [33]. The lipid profiles were not associated with either the genotypes or the alleles of the L162V polymorphism in the PPARα gene (Table 3).

To analyze the association between the genotypic and phenotypic expressions and the outcomes, we performed a multivariate analysis to calculate the adjusted odds ratio (OR) because of the significant differences in the ages and gender distribution among the studied groups. The results of this analysis suggested that the presence of the lipoprotein lipase H+/H+ genotype (adjusted OR = 21.3; 95% CI = 2.32–335.3; *P* = 0.003), the PPARα Leu/Val genotype (adjusted OR = 8.77; 95% CI = 1.41–78.7; *P* = 0.014), and elevated levels of VLDL-C (adjusted OR = 1.08; 95% CI = 1.01–1.17; *P* = 0.023) and TAG (adjusted OR = 1.02; 95% CI = 1.00–1.03; *P* = 0.021) were associated with symptomatic* L. infantum* infection. In contrast, the HDL levels were inversely associated with symptomatic (adjusted OR = 0.84; 95% CI = 0.71–0.96; *P* = 0.006) and asymptomatic (adjusted OR = 0.92; 95% CI = 0.84–0.99; *P* = 0.027)* L. infantum* infection (Table 4). When the lipoprotein levels were analyzed using the cut-off value established by selecting the point on the ROC curve that maximized the sensitivity and specificity for separating noninfected and infected symptomatic individuals, the association with symptomatic infection became more evident for elevated levels of VLDL-C (cut-off value = 33 mg/dL; adjusted OR = 18.8; 95% CI = 1.71–1014.4; *P* = 0.009) and TAG (cut-off value = 168 mg/dL; adjusted OR = 18.8; 95% CI = 1.71–1014.4; *P* = 0.009).

## 4. Discussion

Discovering reliable markers for the assessment of individual and population risks of disease development is a major challenge for the scientific community. In this study, we evaluated potential markers of VL development. In areas that are endemic for VL, most infected individuals develop a protective immune response. Therefore, we focused on the nonimmune lipid elements as potential susceptibility factors based on the hypothesis that these alterations in VL patients may favor parasite growth and disease development. We further analyzed the polymorphisms of certain genes that may lead to such alterations in lipoprotein levels.

In a significant number of patients with clinical manifestations of VL, we found high TAG levels, high VLDL-C levels, and low HDL-C levels in infected symptomatic individuals as previously reported in the literature [19–22]. LPL, apoE, and PPARα are known as important components of lipid metabolism with roles in the homeostasis of different lipoprotein fractions and their transport, and the polymorphisms of these genes have been associated with cardiovascular diseases [28, 29, 34–36]. Therefore, these gene polymorphisms were analyzed in this study. We targeted LPL, apoE, and PPARα genes with polymorphisms and alleles that were analyzed in relation to symptomatic* L. infantum* infection. Importantly, an association between these polymorphisms and the expected phenotypes was observed, excluding the L162V polymorphism in the PPARα gene. This finding is in contrast to most studies on gene polymorphisms in which the resulting phenotypes were unidentified. In the studied population, the H+/H+ genotype and the H+ allele were associated with elevated VLDL-C and TAG levels (*P* < 0.05) and reduced HDL-C levels (*P* < 0.05). For the apoE genes and alleles, the genotype and expected phenotype association was unclear because the TAG and VLDL-C levels were significantly higher in individuals with the E2 allele than in individuals with the E3 and E4 alleles but not in individuals with the E3 allele. This finding may be due to the restricted number of samples and to the different roles of the six apoE allotypes. However, the association between the E2 allele and the alterations in lipoprotein levels may not have been uncovered because apoE2 carries significantly less VLDL cholesterol than apoE4 [35], and apoE2 impairs the lipolytic conversion of VLDL to LDL through inhibiting LPL activity [34].

The association between the H+/H+ genotype and the H+ allele with higher TAG and lower HDL-C levels was stronger in the infected symptomatic group than in the infected asymptomatic group. PvuII has been associated with variations in serum lipids in other diseases [29, 36]; however, we did not detect any significant associations between the PvuII-restricted LPL genotypes and the presence of active VL or lipid fraction levels. This finding reinforces our suggestion that the HindIII-restricted LPL genotypes that were identified in this study are risk markers for the development of symptomatic* L. infantum* infection.

In the literature, high TAG and low HDL-C levels have been suggested as parameters for the diagnosis and follow-up of patients with VL [37]. However, in addition to a single study that suggested a role for LDL in the development of VL through tumor necrosis factor-α (TNF-α) production [22], our study is the first to clearly find that the LPL genotype that leads to a phenotype of high VLDL-C levels, high TAG levels, and low HDL-C levels is a risk factor for active visceral leishmaniasis. Interestingly, we observed reduced high-density lipoprotein cholesterol (HDL-C) levels in both the infected symptomatic and asymptomatic individuals compared with the noninfected individuals, which may suggest that decreased HDL levels initially favor infection. When TG and VLDL levels increase, progression to symptomatic infection may occur.

The biological roles of VLDL and HDL in* Leishmania* infection need to be further analyzed; however, as an initial approach, we assessed the role of lipoprotein fractions in the mechanism of cell infection in vitro by examining the phagocytosis of promastigotes by macrophages. We observed 29% more parasites within the macrophages that were incubated for six hours with VLDL (*P* < 0.05) than in culture without the addition of lipoprotein. The coaddition of HDL significantly reduced (37%) the parasite burden compared with VLDL (*P* < 0.05).

The PPARα L162V polymorphism has been demonstrated to be a risk marker for symptomatic infection; however, the mutated gene was not associated with altered lipoprotein levels. In a functional study of the Val-mutated PPARα allele, this mutation in the DNA binding domain for PPARα impacted the function of this transcription factor by preventing its proper binding to the region of DNA that is under its transcriptional control [38]. The frequency of this mutated allele was significantly higher in individuals who presented with symptomatic infection; therefore, the inefficient activation of lipid metabolism genes may lead to higher serum levels of lipid fractions. However, the results of this study did not indicate a relationship between the polymorphisms and the lipid profiles. A possible explanation for this finding is the low number of individuals who presented with the Val allele. This variant allele is relatively rare (an observed frequency of 10.8%), and the lipid profiles of only 8 individuals who were carrying this allele were measured. Therefore, any statistical evidence for a relationship between this mutation and the lipid profiles would be unlikely. Besides the PPARα gene can modulate lipid pathways, which may not be reflected in serum-lipid levels, and indirectly affect the outcome of* Leishmania infantum* infection. In addition, PPARα is known for its major role in the upregulation of cholesterol trafficking and efflux in macrophages [39]. Interestingly, in transcriptomic studies with* Leishmania*-infected mouse macrophages, cholesterol accumulation was clearly observed within host cells, which was achieved by the upregulation of genes that are implicated in the uptake of LDL (CD36 and the LDL receptor) and by the downregulation of genes that mediate cholesterol efflux (ABCA1 and CYP27) [40, 41]. In this scenario, a PPARα variant allele may contribute to the modulation of infection by enhancing the accumulation of cholesterol in infected cells and by shuttling cholesterol to* Leishmania* parasites. We cannot rule out either the modulation of infection by PPARα functions in inflammatory processes [39].

The results of this study and those from two previous studies [30, 42] support the presupposition that the genotypes for molecules that are not related to the adaptive immune system, that is, LPL, PPARα, and mannan-binding lectin (MBL) genotypes, have a significant correlation with* L. infantum *infection or disease. Notably, in addition to the roles of MBL in the complement system [43] and the autoimmune process [44], MBL levels are affected by the activation of PPARα [45], which induces LPL expression [46].

These genetic markers and the more easily accessible lipid levels may be used to evaluate the risk of* L. infantum* infection and disease in endemic areas and may be used to follow up patients who present with active-phase infection. Furthermore, our results suggest that we should consider studying lipid-lowering drugs as a complementary treatment for VL patients.

## Figures and Tables

**Table 1:** Levels of serum lipids in individuals exposed to *Leishmania infantum* from endemic area.

Lipids	Infected symptomatic individuals		Infected asymptomatic individuals		Noninfected individuals		*P* value
	*n*	mg/dL	*n*	mg/dL	*n*	mg/dL	
TC	17	121.2 ± 34.6	28	143.0 ± 42.1	36	139.9 ± 36.1	0.152
LDL-C	17	59.5 ± 30.8	28	85.5 ± 42.2	36	77.1 ± 36.0	0.083
HDL-C	17	38.3 ± 4.3∗	28	39.0 ± 5.8∗	36	43.0 ± 7.2	**0.012**
TAG	17	219.4 ± 84.9^∗,†^	28	169.1 ± 62.7	36	157.9 ± 67.6	**0.013**
VLDL-C	17	43.8 ± 16.9^∗,†^	28	33.9 ± 12.4	36	31.6 ± 13.5	**0.013**

TC = total cholesterol; LDL-C = low-density lipoprotein cholesterol; HDL-C = density lipoprotein cholesterol; TAG = triacylglycerol; VLDL = very-low-density lipoprotein cholesterol. Lipid values expressed as the mean ± SD. *P* value analyzed by ANOVA test with the Student Newman-Keuls posttest. *P < 0.05 versus noninfected individuals; ^†^ *P* < 0.05 versus infected asymptomatic individuals. *P* values in bold indicate statistically significant differences.

**Table 2:** Frequency of genotypes and alleles of LPL, apoE, and PPAR*α* genes in individuals exposed to *Leishmania infantum*.

	Polymorphisms^§§^	Infected symptomatic individuals		Infected asymptomatic individuals		Noninfected individuals		*P* value
		*n*	% of total	*n*	% of total	*n*	% of total	
HindIII	H+/H+^(a)^	24	46.2	17	35.4	13	23.2	0.040^(a vs. b)^
	H+/H−^(b)^	26	50.0	27	56.3	34	60.7	0.103^(b vs. c)^
	H−/H−^(c)^	2	3.8	4	8.3	9	16.1	0.006^(a vs. c)^
		HWE^†^	*0.116 *		*0.138 *		*0.099 *	
	H+^(d)^	74	71.5	61	63.5	60	53.6	
	H−^(e)^	30	28.8	35	36.5	52	46.4	0.008^(d vs. e)^

PvuII	P1/P1^(f)^	12	28.6	13	29.5	16	37.2	0.429^(f vs. g)^
	P1/P2^(g)^	25	59.5	27	61.4	23	53.5	0.835^(g vs. h)^
	P2/P2^(h)^	5	11.9	4	9.1	4	9.3	0.507^(f vs. h)^
		HWE^†^	*0.146 *		*0.062 *		*0.294 *	
	P1^(i)^	49	58.3	53	60.2	55	64.0	
	P2^(j)^	35	41.7	35	39.8	31	36.0	0.452^(i vs. j)^

ApoE	E2/E3^(k)^	3	8.1	4	10.5	2	5.0	0.833^(k vs. l)^
	E3/E3^(l)^	30	81.1	24	63.2	26	65.0	0.116^(l vs. m)^
	E3/E4^(m)^	4	10.8	10	26.3	10	25.0	0.214^(k vs. m)^
	E2/E4	0	0.0	0	0.0	1	2.5	
	E4/E4	0	0.0	0	0.0	1	2.5	
	E2^(n)^	3	4.1	4	5.3	3	3.7	0.953^(n vs. o)^
	E3^(o)^	67	90.5	62	81.6	64	80.0	0.038^(o vs. p)^
	E4^(p)^	4	5.4	10	13.1	13	16.3	0.230^(n vs. p)^

PPPAR*α*	Leu/Leu^(q)^	48	80.0	61	87.1	92	93.9	**0.022** ^(q vs. r)^
	Leu/Val^(r)^	11	18.3	8	11.4	6	6.1	
	Val/Val	1	1.7	1	1.4	0	0.0	
		HWE^†^	*0.693 *		*0.247 *		*0.745 *	
	Leu^(s)^	107	89.2	130	92.9	190	96.9	
	Val^(t)^	13	10.8	10	7.1	6	3.1	0.008^(s vs. t)^

^§§^ *P* value analyzed by *χ* ^2^ test for trend; ^†^ *P* value in Hardy-Weinberg equilibrium (HWE).

Note: H+ indicates presence and H− indicates absence of the HindIII restriction site. P1 indicates absence and P2 indicates presence of the PvuII restriction site. Apo E polymorphism results in six genotypes (E2/E2, E2/E3, E2/E4, E3/E3, E3/E4, and E4/E4). E2/E4 and E4/E4 did not have sufficient number to be analyzed. E2/E2 was not detected. PPAR*α* L162V polymorphism results in three genotypes (Leu/Leu, Leu/Val, and Val/Val). Bold text indicates statistically significant differences.

**Table 3:** Levels of lipids (mg/dL) according to genotypes and alleles of LPL and of apoE in individuals exposed to *Leishmania infantum*.

		Polymorphisms	Age	Lipids (mg/dL)				
				TC^a^	LDL-C^a^	HDL-C^a^	TAG^a^	VLDL-C^a^
HindIII	Genotypes	H+/H+ (*n* = 19)	22.6 ± 15.4	150.8 ± 42.2	91.0 ± 44.0	38.1 ± 5.6	207.6 ± 82.7	41.5 ± 16.5
		H+/H− (*n* = 33)	22.1 ± 18.2	138.8 ± 34.8	77.4 ± 35.6	41.9 ± 6.4	157.9 ± 52.8	31.6 ± 10.5
		*P* value^#^	0.914	0.274	0.228	**0.037**	**0.011**	**0.010**
	Alleles	H+ (*n* = 71)	22.4 ± 16.6	145.3 ± 38.8	84.7 ± 40.3	39.9 ± 6.2	184.5 ± 73.6	36.9 ± 14.6
		H− (*n* = 37)	22.7 ± 17.5	135.2 ± 34.7	74.4 ± 34.7	42.5 ± 6.3	154.5 ± 50.8	30.9 ± 10.1
		*P* value^#^	0.919	0.187	0.191	**0.040**	**0.029**	**0.029**

PvuII	Genotypes	P1/P1 (*n* = 15)	22.4 ± 14.0	24.6 ± 16.7	81.7 ± 37.1	40.0 ± 4.7	159.4 ± 50.9	31.9 ± 10.1
		P1/P2 (*n* = 27)	24.6 ± 16.7	141.8 ± 38.7	84.3 ± 39.7	39.9 ± 6.3	173.0 ± 77.7	34.6 ± 15.5
		P2/P2 (*n* = 4)	16.5 ± 17.8	132.8 ± 40.3	71.7 ± 28.8	41.5 ± 5.7	230.5 ± 89.0	46.0 ± 17.6
		*P*value^§^	0.622	0.878	0.828	0.878	0.217	0.220
	Alleles	P1 (*n* = 57)	23.5 ± 15.1	142.8 ± 36.5	82.9 ± 37.7	40.0 ± 5.4	165.8 ± 64.4	165.8 ± 64.4
		P2 (*n* = 35)	22.8 ± 16.7	139.7 ± 38.1	81.4 ± 37.1	40.3 ± 6.1	186.1 ± 81.4	37.2 ± 16.2
		*P* value^#^	0.840	0.706	0.853	0.793	0.188	0.191

apoE	Genotypes	E2/E3 (*n* = 4)	23.7 ± 14.3	120.0 ± 36.2	65.7 ± 30.4	41.7 ± 7.8	275.3 ± 113.5^∗,†^*l*^^	54.7 ± 22.7^∗,†^
		E3/E3 (*n* = 29)	19.1 ± 14.8	152.1 ± 40.1	91.5 ± 41.6	39.2 ± 5.8	166.9 ± 58.5	33.4 ± 11.6
		E3/E4 (*n* = 6)	35.5 ± 18.1	119.0 ± 11.9	65.7 ± 23.9	39.0 ± 3.0	177.3 ± 100.4	35.5 ± 19.9
		*P*value^§^	0.068	0.069	0.205	0.692	**0.026**	**0.028**
	Alleles	E2 (*n* = 4)	23.7 ± 14.3	120.0 ± 36.2	65.7 ± 30.4	41.7 ± 7.8	275.3 ± 113.5^∗,†^*l*^^	54.7 ± 22.7^∗,†^
		E3 (*n* = 68)	20.8 ± 15.5	147.3 ± 39.4^†,‡^	87.7 ± 40.2	39.2 ± 5.7	174.2 ± 69.6	34.8 ± 13.8
		E4 (*n* = 8)	32.1 ± 16.5	113.0 ± 15.0	59.5 ± 23.2	38.7 ± 2.6	165.5 ± 87.7	33.1 ± 17.4
		*P*value^§^	0.152	**0.029**	0.099	0.661	**0.029**	**0.031**

PPAR*α*	Genotypes	Leu/Leu (*n* = 54)	26.7 ± 17.6	137.9 ± 39.7	78.2 ± 38.8	40.9 ± 6.5	173.2 ± 81.2	34.6 ± 16.2
		Leu/Val (*n* = 8)	23.1 ± 18.3	146.9 ± 32.6	83.3 ± 31.1	40.3 ± 5.7	166.1 ± 35.7	33.4 ± 6.9
		*P* value^#^	0.589	0.545	0.728	0.794	0.809	0.830
	Alleles	Leu (*n* = 116)	26.5 ± 17.5	138.5 ± 39.0	78.6 ± 38.1	40.8 ± 6.4	172.7 ± 78.5	34.5 ± 15.6
		Val (*n* = 8)	23.1 ± 18.3	146.9 ± 32.6	83.3 ± 31.1	40.3 ± 5.7	166.1 ± 35.7	33.4 ± 6.9
		*P* value^#^	0.599	0.555	0.735	0.798	0.814	0.834

All values are expressed as the mean ± SD in individuals exposed to *Leishmania chagasi*.

^a^values in mg/dL. ^#^ *P* value analyzed by Student *t*-test; ^§^ *P * value analyzed by ANOVA test with the Student Newman-Keuls posttest. In relation to genotypes: *P < 0.05 versus E3/E3; ^†^ *P* < 0.05 versus E3/E4. In relation to alleles: *P < 0.05 versus E3; ^†^ *P* < 0.05 versus E4; ^‡^ *P* < 0.05 versus E2. H−/H− did not have sufficient number to be analyzed (*n* = 2). Age = 28.0 ± 12.7; TC = 105.0 ± 14.1; LDL-C = 50.0 ± 7.1; HDL-C = 47.5 ± 2.1; TAG = 126.5 ± 9.2; VLDL-C = 25.5 ± 2.1. E4/E4 did not have sufficient number to be analyzed (*n* = 1). Age = 22.0; TC = 95.0; LDL-C = 41.0; HDL-C = 38.0; TAG = 130.0; VLDL = 26.0. H+ indicates presence and H− indicates absence of the HindIII restriction site. P1 indicates absence and P2 indicates presence of the PvuII restriction site.

Note: Apo E polymorphism results in six genotypes ranking (E2/E2, E2/E3, E2/E4, E3/E3, E3/E4, and E4/E4) and three alleles (E2, E3, and E4). Bold text indicates statistically significant differences.

**Table 4:** Adjusted odds ratios (OR) and respective 95% confidence intervals for the association between levels of lipids (mg/dL) and genotypes of LPL, apoE, and PPAR*α* and infected symptomatic and asymptomatic *L. infantum* infection.

Variable	Infected symptomatic infection			Infected asymptomatic infection		
	OR∗	95% CI∗	*P* value	OR∗	95% CI∗	*P* value
HindIII						
H−/H−	1.00			1.00		
H+/H−	6.37	0.84–83.4	0.083	2.04	0.49–10.4	0.435
H+/H+	21.3	2.32–335.3	**0.003**	3.48	0.72–20.4	0.143
PvuII						
P1/P1	1.00			1.00		
P1/P2	1.86	0.55–6.68	0.395	1.42	0.52–3.98	0.593
P2/P2	0.71	0.10–5.28	1.000	1.20	0.18–7.81	1.000
ApoE						
E3/E4	1.00			1.00		
E3/E3	2.66	0.52–16.1	0.312	0.91	0.28–2.93	1.000
E2/E3	4.31	0.23–113.7	0.515	1.90	0.22 –25.3	0.825
PPAR*α*						
Leu/Leu	1.00			1.00		
Leu/Val	8.77	1.41–78.7	**0.014**	2.76	0.56–17.9	0.272
TC	0.99	0.97–1.01	0.301	1.00	0.99–1.01	0.787
LDL-C	0.99	0.97–1.01	0.353	1.01	0.99–1.02	0.414
HDL-C	0.84	0.71–0.96	**0.006**	0.92	0.84–0.99	**0.027**
TAG	1.02	1.00–1.03	**0.021**	1.00	0.99–1.01	0.597
VLDL-C	1.08	1.01–1.17	**0.023**	1.01	0.97–1.05	0.580

∗Adjusted for age (≤ or >20 years) and gender.

*P* values in bold indicate statistically significant differences.
